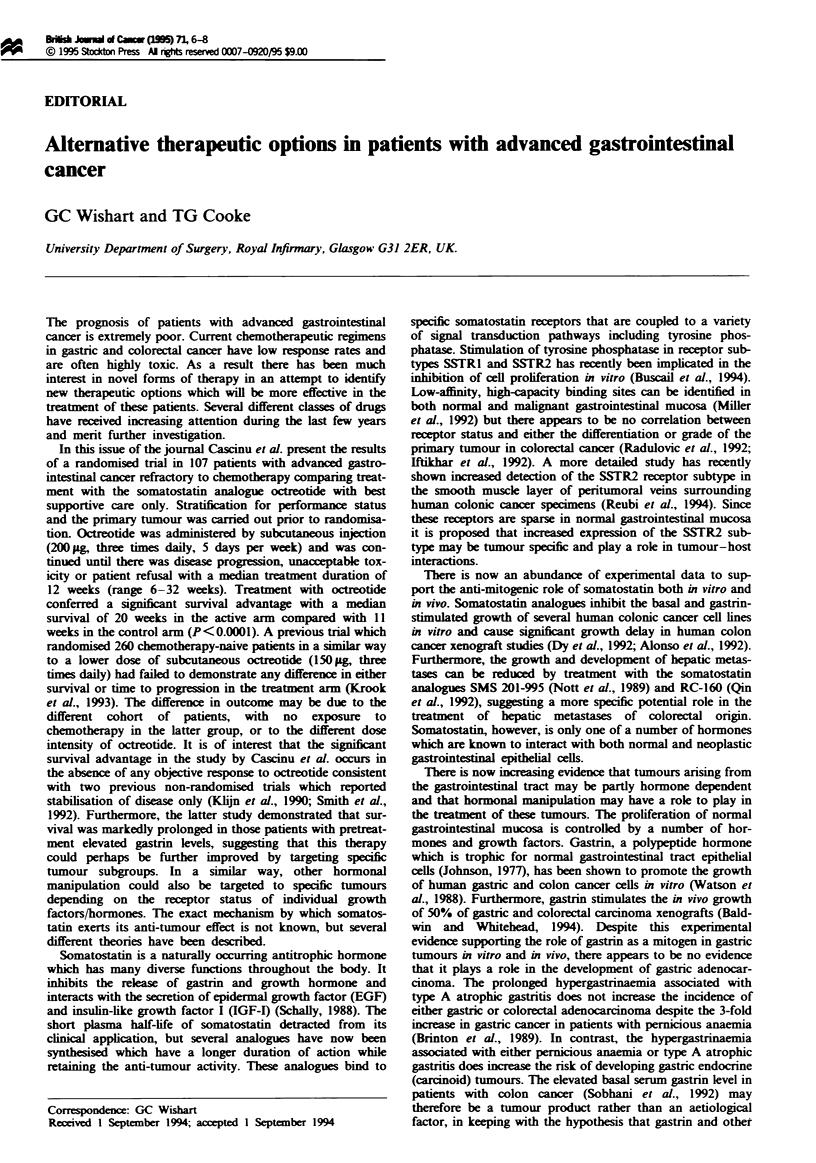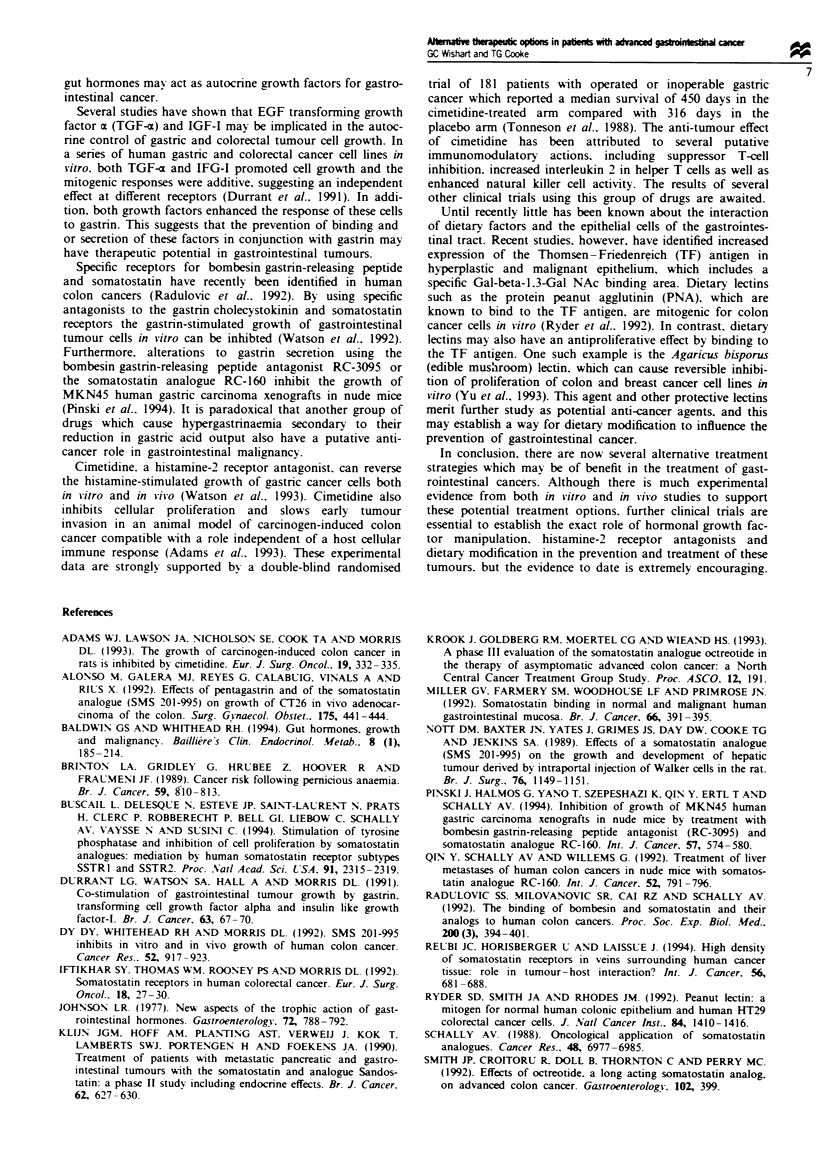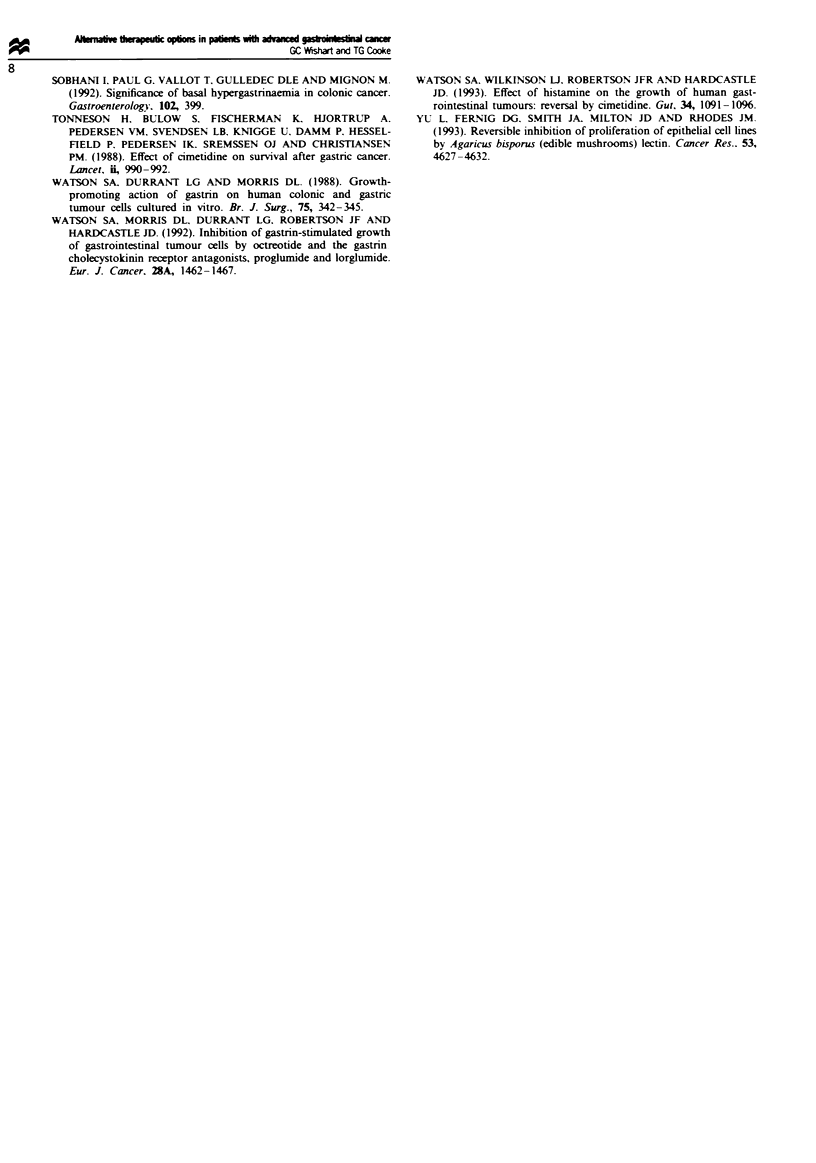# Alternative therapeutic options in patients with advanced gastrointestinal cancer.

**DOI:** 10.1038/bjc.1995.2

**Published:** 1995-01

**Authors:** G. C. Wishart, T. G. Cooke


© 1995 Stockton Press All rights reserved 0007–0920/95 $9.00

EDITORIAL

### Alternative therapeutic options in patients with advanced gastrointestinal cancer

GC Wishart and TG Cooke

*University Department of Surgery, Royal Infirmary, Glasgow G31 2ER, UK.*

The prognosis of patients with advanced gastrointestinal cancer is extremely poor. Current chemotherapeutic regimens in gastric and colorectal cancer have low response rates and are often highly toxic. As a result there has been much interest in novel forms of therapy in an attempt to identify new therapeutic options which will be more effective in the treatment of these patients. Several different classes of drugs have received increasing attention during the last few years and merit further investigation.

In this issue of the journal Cascinu *et al.* present the results of a randomised trial in 107 patients with advanced gastrointestinal cancer refractory to chemotherapy comparing treatment with the somatostatin analogue octreotide with best supportive care only. Stratification for performance status and the primary tumour was carried out prior to randomisation. Octreotide was administered by subcutaneous injection (200 μg, three times daily, 5 days per week) and was continued until there was disease progression, unacceptable toxicity or patient refusal with a median treatment duration of 12 weeks (range 6–32 weeks). Treatment with octreotide conferred a significant survival advantage with a median survival of 20 weeks in the active arm compared with 11 weeks in the control arm ($P < 0.0001$). A previous trial which randomised 260 chemotherapy-naive patients in a similar way to a lower dose of subcutaneous octreotide (150 μg, three times daily) had failed to demonstrate any difference in either survival or time to progression in the treatment arm (Krook *et al.*, 1993). The difference in outcome may be due to the different cohort of patients, with no exposure to chemotherapy in the latter group, or to the different dose intensity of octreotide. It is of interest that the significant survival advantage in the study by Cascinu *et al.* occurs in the absence of any objective response to octreotide consistent with two previous non-randomised trials which reported stabilisation of disease only (Klijn *et al.*, 1990; Smith *et al.*, 1992). Furthermore, the latter study demonstrated that survival was markedly prolonged in those patients with pretreatment elevated gastrin levels, suggesting that this therapy could perhaps be further improved by targeting specific tumour subgroups. In a similar way, other hormonal manipulation could also be targeted to specific tumours depending on the receptor status of individual growth factors/hormones. The exact mechanism by which somatostatin exerts its anti-tumour effect is not known, but several different theories have been described.

Somatostatin is a naturally occurring antitrophic hormone which has many diverse functions throughout the body. It inhibits the release of gastrin and growth hormone and interacts with the secretion of epidermal growth factor (EGF) and insulin-like growth factor I (IGF-I) (Schally, 1988). The short plasma half-life of somatostatin detracted from its clinical application, but several analogues have now been synthesised which have a longer duration of action while retaining the anti-tumour activity. These analogues bind to specific somatostatin receptors that are coupled to a variety of signal transduction pathways including tyrosine phosphatase. Stimulation of tyrosine phosphatase in receptor subtypes SSTR1 and SSTR2 has recently been implicated in the inhibition of cell proliferation *in vitro* (Buscail *et al.*, 1994). Low-affinity, high-capacity binding sites can be identified in both normal and malignant gastrointestinal mucosa (Miller *et al.*, 1992) but there appears to be no correlation between receptor status and either the differentiation or grade of the primary tumour in colorectal cancer (Radulovic *et al.*, 1992; Iftikhar *et al.*, 1992). A more detailed study has recently shown increased detection of the SSTR2 receptor subtype in the smooth muscle layer of peritumoral veins surrounding human colonic cancer specimens (Reubi *et al.*, 1994). Since these receptors are sparse in normal gastrointestinal mucosa it is proposed that increased expression of the SSTR2 subtype may be tumour specific and play a role in tumour–host interactions.

There is now an abundance of experimental data to support the anti-mitogenic role of somatostatin both *in vitro* and *in vivo*. Somatostatin analogues inhibit the basal and gastrin-stimulated growth of several human colonic cancer cell lines *in vitro* and cause significant growth delay in human colon cancer xenograft studies (Dy *et al.*, 1992; Alonso *et al.*, 1992). Furthermore, the growth and development of hepatic metastases can be reduced by treatment with the somatostatin analogues SMS 201-995 (Nott *et al.*, 1989) and RC-160 (Qin *et al.*, 1992), suggesting a more specific potential role in the treatment of hepatic metastases of colorectal origin. Somatostatin, however, is only one of a number of hormones which are known to interact with both normal and neoplastic gastrointestinal epithelial cells.

There is now increasing evidence that tumours arising from the gastrointestinal tract may be partly hormone dependent and that hormonal manipulation may have a role to play in the treatment of these tumours. The proliferation of normal gastrointestinal mucosa is controlled by a number of hormones and growth factors. Gastrin, a polypeptide hormone which is trophic for normal gastrointestinal tract epithelial cells (Johnson, 1977), has been shown to promote the growth of human gastric and colon cancer cells *in vitro* (Watson *et al.*, 1988). Furthermore, gastrin stimulates the *in vivo* growth of 50% of gastric and colorectal carcinoma xenografts (Baldwin and Whitehead, 1994). Despite this experimental evidence supporting the role of gastrin as a mitogen in gastric tumours *in vitro* and *in vivo*, there appears to be no evidence that it plays a role in the development of gastric adenocarcinoma. The prolonged hypergastrinaemia associated with type A atrophic gastritis does not increase the incidence of either gastric or colorectal adenocarcinoma despite the 3-fold increase in gastric cancer in patients with pernicious anaemia (Brinton *et al.*, 1989). In contrast, the hypergastrinaemia associated with either pernicious anaemia or type A atrophic gastritis does increase the risk of developing gastric endocrine (carcinoid) tumours. The elevated basal serum gastrin level in patients with colon cancer (Sobhani *et al.*, 1992) may therefore be a tumour product rather than an aetiological factor, in keeping with the hypothesis that gastrin and other

Correspondence: GC Wishart
Received 1 September 1994; accepted 1 September 1994

gut hormones may act as autocrine growth factors for gastrointestinal cancer.

Several studies have shown that EGF transforming growth factor α (TGF-α) and IGF-I may be implicated in the autocrine control of gastric and colorectal tumour cell growth. In a series of human gastric and colorectal cancer cell lines *in vitro*, both TGF-α and IFG-I promoted cell growth and the mitogenic responses were additive, suggesting an independent effect at different receptors (Durrant *et al.*, 1991). In addition, both growth factors enhanced the response of these cells to gastrin. This suggests that the prevention of binding and or secretion of these factors in conjunction with gastrin may have therapeutic potential in gastrointestinal tumours.

Specific receptors for bombesin gastrin-releasing peptide and somatostatin have recently been identified in human colon cancers (Radulovic *et al.*, 1992). By using specific antagonists to the gastrin cholecystokinin and somatostatin receptors the gastrin-stimulated growth of gastrointestinal tumour cells *in vitro* can be inhibted (Watson *et al.*, 1992). Furthermore, alterations to gastrin secretion using the bombesin gastrin-releasing peptide antagonist RC-3095 or the somatostatin analogue RC-160 inhibit the growth of MKN45 human gastric carcinoma xenografts in nude mice (Pinski *et al.*, 1994). It is paradoxical that another group of drugs which cause hypergastrinaemia secondary to their reduction in gastric acid output also have a putative anticancer role in gastrointestinal malignancy.

Cimetidine, a histamine-2 receptor antagonist, can reverse the histamine-stimulated growth of gastric cancer cells both *in vitro* and *in vivo* (Watson *et al.*, 1993). Cimetidine also inhibits cellular proliferation and slows early tumour invasion in an animal model of carcinogen-induced colon cancer compatible with a role independent of a host cellular immune response (Adams *et al.*, 1993). These experimental data are strongly supported by a double-blind randomised trial of 181 patients with operated or inoperable gastric cancer which reported a median survival of 450 days in the cimetidine-treated arm compared with 316 days in the placebo arm (Tonneson *et al.*, 1988). The anti-tumour effect of cimetidine has been attributed to several putative immunomodulatory actions, including suppressor T-cell inhibition, increased interleukin 2 in helper T cells as well as enhanced natural killer cell activity. The results of several other clinical trials using this group of drugs are awaited.

Until recently little has been known about the interaction of dietary factors and the epithelial cells of the gastrointestinal tract. Recent studies, however, have identified increased expression of the Thomsen–Friedenreich (TF) antigen in hyperplastic and malignant epithelium, which includes a specific Gal-beta-1,3-Gal NAc binding area. Dietary lectins such as the protein peanut agglutinin (PNA), which are known to bind to the TF antigen, are mitogenic for colon cancer cells *in vitro* (Ryder *et al.*, 1992). In contrast, dietary lectins may also have an antiproliferative effect by binding to the TF antigen. One such example is the *Agaricus bisporus* (edible mushroom) lectin, which can cause reversible inhibition of proliferation of colon and breast cancer cell lines *in vitro* (Yu *et al.*, 1993). This agent and other protective lectins merit further study as potential anti-cancer agents, and this may establish a way for dietary modification to influence the prevention of gastrointestinal cancer.

In conclusion, there are now several alternative treatment strategies which may be of benefit in the treatment of gastrointestinal cancers. Although there is much experimental evidence from both *in vitro* and *in vivo* studies to support these potential treatment options, further clinical trials are essential to establish the exact role of hormonal growth factor manipulation, histamine-2 receptor antagonists and dietary modification in the prevention and treatment of these tumours, but the evidence to date is extremely encouraging.

### References

ADAMS WJ, LAWSON JA, NICHOLSON SE, COOK TA AND MORRIS DL. (1993). The growth of carcinogen-induced colon cancer in rats is inhibited by cimetidine. *Eur. J. Surg. Oncol.*, **19**, 332–335.

ALONSO M, GALERA MJ, REYES G, CALABUIG, VINALS A AND RIUS X. (1992). Effects of pentagastrin and of the somatostatin analogue (SMS 201-995) on growth of CT26 in vivo adenocarcinoma of the colon. *Surg. Gynaecol. Obstet.*, **175**, 441–444.

BALDWIN GS AND WHITHEAD RH. (1994). Gut hormones, growth and malignancy. *Baillière's Clin. Endocrinol. Metab.*, **8 (1)**, 185–214.

BRINTON LA, GRIDLEY G, HRUBEE Z, HOOVER R AND FRAUMENI JF. (1989). Cancer risk following pernicious anaemia. *Br. J. Cancer*, **59**, 810–813.

BUSCAIL L, DELESQUE N, ESTEVE JP, SAINT-LAURENT N, PRATS H, CLERC P, ROBBERECHT P, BELL GI, LIEBOW C, SCHALLY AV, VAYSSE N AND SUSINI C. (1994). Stimulation of tyrosine phosphatase and inhibition of cell proliferation by somatostatin analogues: mediation by human somatostatin receptor subtypes SSTR1 and SSTR2. *Proc. Natl Acad. Sci. USA*, **91**, 2315–2319.

DURRANT LG, WATSON SA, HALL A AND MORRIS DL. (1991). Co-stimulation of gastrointestinal tumour growth by gastrin, transforming cell growth factor alpha and insulin like growth factor-I. *Br. J. Cancer*, **63**, 67–70.

DY DY, WHITEHEAD RH AND MORRIS DL. (1992). SMS 201-995 inhibits in vitro and in vivo growth of human colon cancer. *Cancer Res.*, **52**, 917–923.

IFTIKHAR SY, THOMAS WM, ROONEY PS AND MORRIS DL. (1992). Somatostatin receptors in human colorectal cancer. *Eur. J. Surg. Oncol.*, **18**, 27–30.

JOHNSON LR. (1977). New aspects of the trophic action of gastrointestinal hormones. *Gastroenterology*, **72**, 788–792.

KLIJN JGM, HOFF AM, PLANTING AST, VERWEIJ J, KOK T, LAMBERTS SWJ, PORTENGEN H AND FOEKENS JA. (1990). Treatment of patients with metastatic pancreatic and gastrointestinal tumours with the somatostatin and analogue Sandostatin: a phase II study including endocrine effects. *Br. J. Cancer*, **62**, 627–630.

KROOK J, GOLDBERG RM, MOERTEL CG AND WIEAND HS. (1993). A phase III evaluation of the somatostatin analogue octreotide in the therapy of asymptomatic advanced colon cancer: a North Central Cancer Treatment Group Study. *Proc. ASCO*, **12**, 191.

MILLER GV, FARMERY SM, WOODHOUSE LF AND PRIMROSE JN. (1992). Somatostatin binding in normal and malignant human gastrointestinal mucosa. *Br. J. Cancer*, **66**, 391–395.

NOTT DM, BAXTER JN, YATES J, GRIMES JS, DAY DW, COOKE TG AND JENKINS SA. (1989). Effects of a somatostatin analogue (SMS 201-995) on the growth and development of hepatic tumour derived by intraportal injection of Walker cells in the rat. *Br. J. Surg.*, **76**, 1149–1151.

PINSKI J, HALMOS G, YANO T, SZEPESHAZI K, QIN Y, ERTL T AND SCHALLY AV. (1994). Inhibition of growth of MKN45 human gastric carcinoma xenografts in nude mice by treatment with bombesin gastrin-releasing peptide antagonist (RC-3095) and somatostatin analogue RC-160. *Int. J. Cancer*, **57**, 574–580.

QIN Y, SCHALLY AV AND WILLEMS G. (1992). Treatment of liver metastases of human colon cancers in nude mice with somatostatin analogue RC-160. *Int. J. Cancer*, **52**, 791–796.

RADULOVIC SS, MILOVANOVIC SR, CAI RZ AND SCHALLY AV. (1992). The binding of bombesin and somatostatin and their analogs to human colon cancers. *Proc. Soc. Exp. Biol. Med.*, **200 (3)**, 394–401.

REUBI JC, HORISBERGER U AND LAISSUE J. (1994). High density of somatostatin receptors in veins surrounding human cancer tissue: role in tumour–host interaction? *Int. J. Cancer*, **56**, 681–688.

RYDER SD, SMITH JA AND RHODES JM. (1992). Peanut lectin: a mitogen for normal human colonic epithelium and human HT29 colorectal cancer cells. *J. Natl Cancer Inst.*, **84**, 1410–1416.

SCHALLY AV. (1988). Oncological application of somatostatin analogues. *Cancer Res.*, **48**, 6977–6985.

SMITH JP, CROITORU R, DOLL B, THORNTON C AND PERRY MC. (1992). Effects of octreotide, a long acting somatostatin analog, on advanced colon cancer. *Gastroenterology*, **102**, 399.

SOBHANI I. PAUL G. VALLOT T. GULLEDEC DLE AND MIGNON M. (1992). Significance of basal hypergastrinaemia in colonic cancer. *Gastroenterology*, **102**, 399.

TONNESON H. BULOW S. FISCHERMAN K. HJORTRUP A. PEDERSEN VM. SVENDSEN LB. KNIGGE U. DAMM P. HESSELFIELD P. PEDERSEN IK. SREMSSEN OJ AND CHRISTIANSEN PM. (1988). Effect of cimetidine on survival after gastric cancer. *Lancet*, **ii**, 990-992.

WATSON SA. DURRANT LG AND MORRIS DL. (1988). Growth-promoting action of gastrin on human colonic and gastric tumour cells cultured in vitro. *Br. J. Surg.*, **75**, 342-345.

WATSON SA. MORRIS DL. DURRANT LG. ROBERTSON JF AND HARDCASTLE JD. (1992). Inhibition of gastrin-stimulated growth of gastrointestinal tumour cells by octreotide and the gastrin cholecystokinin receptor antagonists, proglumide and lorglumide. *Eur. J. Cancer*, **28A**, 1462-1467.

WATSON SA. WILKINSON LJ. ROBERTSON JFR AND HARDCASTLE JD. (1993). Effect of histamine on the growth of human gastrointestinal tumours: reversal by cimetidine. *Gut*, **34**, 1091-1096.

YU L. FERNIG DG. SMITH JA. MILTON JD AND RHODES JM. (1993). Reversible inhibition of proliferation of epithelial cell lines by *Agaricus bisporus* (edible mushrooms) lectin. *Cancer Res.*, **53**, 4627-4632.